# Phytosterol Composition of *Arachis hypogaea* Seeds from Different Maturity Classes

**DOI:** 10.3390/molecules24010106

**Published:** 2018-12-29

**Authors:** Wenxu Zhou, William D. Branch, Lissa Gilliam, Julie A. Marshall

**Affiliations:** 1 Department of Chemistry and Biochemistry, Texas Tech University, Lubbock, TX 79409, USA; wenxu.zhou@ttu.edu; 2 Crop & Soil Sciences, University of Georgia, Tifton, GA 30602, USA; wdbranch@uga.edu; 3 Biochemical Research Lab, Lubbock Christian University, Lubbock, TX 79407, USA; lissa.gilliam@lcu.edu; 4 Department of Chemistry and Biochemistry, Lubbock Christian University, Lubbock, TX 79407, USA

**Keywords:** phytosterols, mesocarp, oilseed, maturity, pod-blast, α-tocopherol, oil bodies, campesterol, stigmasterol, β-sitosterol

## Abstract

The seeds of cultivated peanut, *Arachis hypogaea*, are an agronomically important crop produced for human nutrition, oilseed and feed stock. Peanut seed is the single most expensive variable input cost and thus producers require seed with excellent performance in terms of germination efficiency. During the maturation process, triglycerides are stored in oil bodies as an energy resource during germination and seedling development. The stability of oil body membranes is essential for nutrient mobilization during germination. This study focused on evaluating the phytosterol composition in seed components including the kernel, embryo (heart), and seed coat or skin. Samples of different maturity classes were analyzed for macronutrient and phytosterol content. The three biosynthetic end products in the phytosterol pathway, β-sitosterol, campesterol and stigmasterol, comprised 82.29%, 86.39% and 94.25% of seed hearts, kernels and seed coats, respectively. Stigmasterol concentration was highest in the seed kernel, providing an excellent source of this sterol known to have beneficial effects on human health. Peanut hearts contained the highest concentration of sterols by mass, potentially providing protection and resources for the developing seedling. The amount of α-tocopherol increases in peanut hearts during the maturation process, providing protection from temperature stress, as well as stability required for seedling vigor. These results suggest that phytosterols may play a significant role in the performance of seeds, and provide a possible explanation for the poor germination efficiency of immature seeds.

## 1. Introduction

The seeds of cultivated peanut, *Arachis hypogaea*, store proteins, lipids and starch required for energy and growth upon germination. The seed can be harvested to serve as human nutrition, stock feed and biofuels [1,2,3]. Peanuts and other legumes have the ability to fix nitrogen and thus increase the sustainability of agricultural systems [4]. The pressure for peanut seed as plant-based protein and oilseed source is increasing with a growing world population. Due to its agronomic importance, improvement of peanut seed performance is necessary to meet the demand.

Lipid (oil) is the predominant macro component, and generally increases as the peanut seed matures [5]. For mature peanuts, total oil was reported to average about 50% on a fresh weight basis [6]. Oilseeds, such as peanuts, store most of their lipids in small, intracellular organelles commonly called oil bodies [7,8]. Triglycerides form the majority of these oil bodies, and the interior triglycerides are encapsulated by a phospholipid bilayer and embedded oleosin protein. The oil bodies provide a stable energy reserve that can be accessed upon germination [9].

Phytosterols are a special class of structural lipids that provide stability and fluidity in cell membranes including specialized encapsulation of lipids, such as oil bodies [10]. The primary function of phytosterols are as membrane reinforcers and precursors for brassinosteroids, an important phytohormone in plants. Unlike animal and fungi counterparts with cholesterol and ergosterol as the dominant sterol, the plant usually synthesizes an array of sterols with different alkyl group substitutions at the sterol side chain. In addition to the primary function, the correct sterol composition is necessary for many aspects of plant biology such as embryonic pattern formation, cell division, cell elongation, cell polarity, cellulose accumulation, and interactions with other signaling pathways [11].

Physiological maturity impacts seed quality through a variety of mechanisms including desiccation tolerance, preparation of storage reserves and establishment of dormancy [12]. Thus, the impact of seed maturity on germination efficiency is of primary importance to peanut producers. To best assess physiological maturation, a method of classification based on color and morphological differences of the mesocarp was described for determining the developmental stages of fresh peanut [13]. Maturity determination by this method requires removal of a portion of the exocarp to expose the pod mesocarp. The characteristic darkening of the pericarp is part of a progressive change in colors resulting in mesocarp colors from white (immature) to black (mature) [14]. The outer layer of the pod can be removed, and seeds sorted by maturity class (white, yellow, orange, brown or black) in a nondestructive manner allowing for physiological and chemical studies pertaining to the stage of development [12].

Seed maturity proceeds with the thickening of cell walls and addition of oil bodies [15]. For mature peanuts of the highest classification (black mesocarp), the cytoplasm of the parenchyma cells is essentially full of oil bodies. The specific aim of this project seeks to clarify the role of phytosterols in the formation of oil bodies as the seed matures. To accomplish this aim, seeds were organized into different maturity classes and the phytosterols extracted and identified. It is hypothesized that establishment of a critical mass of phytosterols provides the membrane stability for proliferation of oil bodies during maturation.

## 2. Results

### 2.1. Stage of Peanut Maturity and Macronutrient Composition in Different Maturity Classes

Peanut seed development can be classified into seven classes with four incremental stages in each class [13]. Based on the color of the mesocarp, the last three classes are described and named as “orange”, “brown” or “black”. Pooled samples from each maturity classes were analyzed to determine macronutrient percentages, and results are summarized in Table 1. The data reported is a check for comparison against the Biochemistry Research Laboratory database of crop samples from 2011–2018. The reported results in Table 1 are consistent with observed trends in peanut crop analysis conducted by the Biochemistry Research Laboratory at Lubbock Christian University (LCU) during the 2011–2018 CYs. The USDA National Nutrient Database for Standard Reference (Release 27, Basic Report 16087) reports macronutrient composition as 49.2% fat, 25.8% protein and 4.7% sugars. The reported results are within expected ranges as compared to the standard values. Any slight variation could be attributed to a specific variety versus average trends across all market types of cultivated peanut for a specific crop year.

### 2.2. Isoprenoids and Phytosterol Composition in Different Tissues

**Figure 1:**
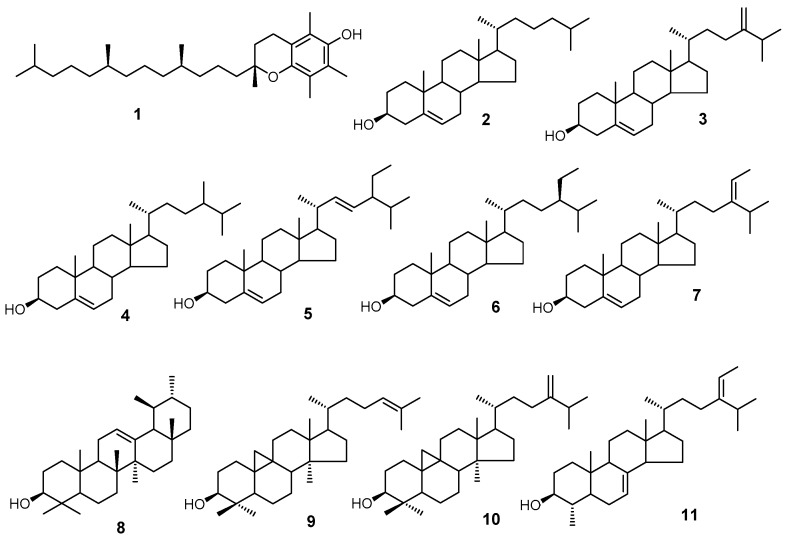
Structures of compounds identified by GC/MS. The structures are **1**. α-tocopherol; **2**. cholesterol; **3**. 24-methylenecholesterol; **4**. campesterol; **5**. stigmasterol; **6**. sitosterol; **7**. isofucosterol; **8**. α-amyrin; **9**. cycloartenol; **10**. 24-methylenecycloartanol; and **11**. citrostadienol.

Using GC/MS, we analyzed three classes of peanut samples for their isoprenoid and sterol composition. We have positively identified 11 compounds (Figure 1) based on the mass spectra and retention time relative to the authentic standards. 

**Figure 2:**
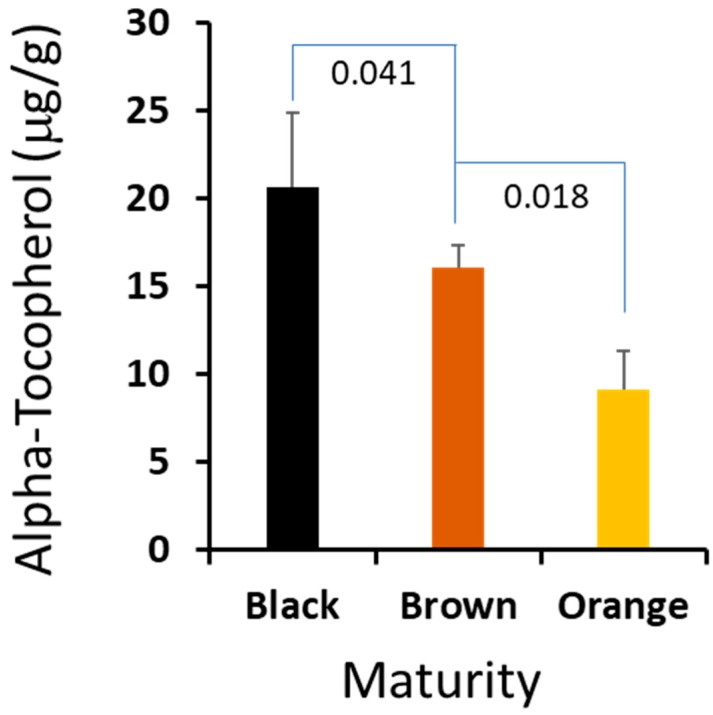
The α-tocopherol contents in the peanuts’ hearts are correlated to the maturity. The error bars are standard deviation of 5 independent measurements and the *p*-values of Student’s *t*-test between the maturity stages.

We found that of the three seed component classes analyzed, the kernel and seed coat generally contained the same percentage of α-tocopherol (0.63% and 0.64%, respectively), decreasing slightly with maturity in brown versus black mesocarp classes, 0.69% to 0.63% kernel composition and 0.71% to 0.64% seed coat composition (Figure 2 and Table 2, Table 3 and Table 4). In contrast, the hearts contained significantly less tocopherol by percentage (Table 2, Table 3, Table 4 and Table 5) as compared to the kernel and seed coat. Also, the amount of α-tocopherol increased from 0.09% in the orange mesocarp class, to 0.19% in the brown mesocarp class, and 0.24% in the black mesocarp class. Vitamin E comprises eight structurally related molecules including four forms of tocopherols. Peanuts, like many other oilseeds, contain tocopherols [16]. Within plants, these molecules are found in cell membranes and possess antioxidant activity which protects organelles from reactive oxygen species (ROS) [17]. Like phytosterols, tocopherols contribute to maintaining membrane fluidity [17]. 

Many phytosterols are present in nature [18]. In plants, the three biosynthetic end products in the phytosterol pathway are β-sitosterol, campesterol and stigmasterol. In 2004, it was reported that these three sterols account for approximately 95% of total peanut sterols [18]. Our analysis confirmed that these sterols are major components of total peanut sterols in each component, 82.29% of hearts, 86.39% of kernels and 94.25% seed coats (Table 2, Table 3 and Table 4), but at lower percentages as compared to the 2004 reported values. Other minor component phytosterols were detected (Table 2, Table 3 and Table 4) including those not previously identified most likely due to advances in technology. Squalene and γ-tocopherol as reported by Maguire et al. [18] were not detected. This discrepancy of results can be explained in addition to sample variations by two possible explanations. Firstly, squalene is the substrate for phytosterol, whereas γ-tocopherol can be converted to α-tocopherol via a methylation reaction. Depending on the harvest time, these two compounds may be converted to their final products and therefore are undetectable. Secondly, in the references, these sterols and tocopherols were determined by an HPLC method. Because of the differences in UV absorption between the two classes of compounds, tocopherol can be easily overestimated. Also, there were no internal standards used in the literature to correct the extraction and HPLC injection errors, as well as a reference for quantitation. Some variation may also be due to varietal differences, as these samples were isolated from Runner-type peanuts as compared to Spanish, Valencia and Virginia market types.

When comparing sterol composition in the different seed components, the relative percentage of stigmasterol should be noted (Table 2, Table 3 and Table 4), heart 2.80%, kernel 11.31% and seed coat 4.09%. This result differs from the reported value of stigmasterol in Runner peanuts as 11.0% [18]. However, the kernel is the largest component of the seed by mass, and as a result, would more closely reflect data on the entire seed.

### 2.3. Isoprenoid and Phytosterol Composition in Different Maturity Classes

It is critical for farmers to harvest peanut at the optimized maturity to maximize the crop value, and a suitable biomarker could help farmers to harvest at the right time. We compared the profiles of the sterols and isoprenoids from the three stages, and there are no statistically significant differences among the major sterols. However, we found that α-tocopherol in the peanut hearts changed dramatically crossing the maturation stages. The absolute amount of this important metabolite was increased correspondingly from 0.92 μg/mg in orange to 1.61 μg/mg in brown, and peaked at 2.08 μg/mg in black. Given the fact that vitamin E is a very important metabolite to plant biology and a valuable compound to human health, we think that the content of vitamin E could be used as a biomarker for peanut harvesting. We plan to develop a user-friendly and portable method to determine vitamin E content, which may have important practical value to peanut farmers.

## 3. Discussion

Peanuts are a nutrient rich plant-based protein source that contain vitamins, minerals, antioxidants and bioactive phytochemicals, leading to the perception that peanuts are a “super food” [19]. Peanut phytosterols have been shown to help lower LDL cholesterol by competing in the digestive tract with cholesterol and preventing absorption [20]. One of the major phytosterols, stigmasterol, has been investigated for its pharmacological importance as an antihypercholesterolemic, anti-inflammatory, antioxidant, hypoglycemic and antitumor effector [21,22]. In this study, it is reported that the kernel contains a higher percentage of stigmasterol as compared to the heart and seed coat. Different manufacturing processes may remove the seed coat or heart, so it is beneficial to the health of the consumer that the kernel possess relatively high concentrations of stigmasterol.

In addition to potential health benefits, stigmasterol is thought to play a role in temperature stress tolerance in plants [23]. Drought and extreme heat in the growing season can increase the sensitivity of the plant to opportunistic organisms [24]. Accumulation of critical phytosterols, such as stigmasterol, during pod development may set the foundation for physiological maturation processes and resistance to stress.

Tocopherol content can vary with environmental stress and growing location in addition to other factors [25]. In this study, hearts contained the lowest percentage of α-tocopherol as compared to the other seed components, but the amount increased during maturation. During germination, the stability of the peanut heart, or embryo, is critically important to the development of the seedling and stand establishment under adverse environmental conditions [17]. α-tocopherol deactivates ROS generated during photosynthesis and is upregulated during stressful events [17]. Immature peanut seed is less resistant to stress, and as a result, is more likely to be adversely affected during germination. The results suggest that the synthesis and accumulation of α-tocopherol in developing peanut hearts may be vitally important to seedling vigor upon germination.

## 4. Materials and Methods

### 4.1. Materials and Reagents

Epicoprostanol, 5β-cholestan-3α-ol, heptane (99%), anhydride pyridine (99.9%), and *N*,*O*-bis(trimethylsilyl)trifluoroacetamide (BSTFA) (99.9%) were purchased from Sigma-Aldrich (St. Louis, MO, USA). HPLC-grade *n*-hexane, HPLC-grade methanol, potassium hydroxide (85%) (KOH), acetone, and HPLC-grade dichloromethane were purchased from Thermo Fisher (Waltham, MA, USA).

### 4.2. Pod Blasting

Freshly harvested pods, 2017 crop year (CY), from one specific genotype of runner market-type peanuts grown under conventional cultural practices were obtained from the University of Georgia research facility under the direction of Dr. W.D. Branch. Pods were removed from the plant material, and ‘pod blasted’ to reveal the mesocarp. Pod blasting is a process by which in-shell peanut pods are placed in a wire basket and a residential-style pressure washer is used to spray the shell exterior with high-pressure water, removing the outer portion of the peanut hull and exposing the colored mesocarp layer underneath. The blasted pods were separated by color into three different maturity classes, orange, brown and black. After separation, the remainder of the pod outer layer was removed, and the seeds segregated for additional chemical analyses. Upon receipt at the research laboratory at LCU, samples were examined and verified to be *A. hypogaea* runner type seeds by technicians under the supervision of Dr. Julie Marshall.

### 4.3. Isolation of Peanut Seed Components

For each maturity class, 10–50 g of redskin kernels were weighed and dried in a forced-air oven (VWR, Radnor, PA, USA) at 130 °C for 45 min. After cooling to ambient temperature, the kernels were manually separated by removing the skin, breaking open the seed, and removing the heart to subdivide the samples into three subsections consisting of seed coats, hearts and kernels. The subsections were scaled at a specific mass for 5 replicates. Each replicate of the heart and kernel contained 50 mg while the seed coat replicates contained 20 mg. Each scaled replicate was placed in a 2 mL microcentrifuge tube with locking lid.

### 4.4. Fat, Protein and Sugar Analysis

To analyze the seed sample for total fat by organic solvent extraction, the exact mass of 10 g ± 0.1 g sample was recorded and the sample pre-dried in a forced-air oven at 130 °C for 45 min to remove moisture. After cooling to ambient temperature, the dried sample was quantitatively transferred to an explosion-proof blender jar (Eberbach Corporation, Belleville, MI, USA). 60 mL dichloromethane (DCM) was added and the mixture blended at high speed for 1 min. After allowing the blender jar to cool for 30 s before opening, we removed the blender lid and washed down the sides of the blender with DCM in a wash bottle. We replaced the lid and blended at high speed for an additional 1 min. We allowed the blender jar to cool for 30 s before opening and washing down the sides of the blender with DCM a second time to remove all residue. We carefully poured the blender contents into a Büchner funnel vacuum filter apparatus with a Toxicity Characteristics Leaching Procedure (TCLP) glass fiber filter, rinsing the blender jar residue with DCM into the funnel until all residue was removed. We filtered the mixture, and transferred the filtrate from the vacuum flask to a tared stainless beaker, rinsing the vacuum flask with DCM into the stainless beaker to ensure all residue was transferred. We evaporated the solvent in the stainless flask over a steam bath until all solvent had been removed. We monitored evaporation and weighed the beaker/remaining oil as needed by removing the beaker from the steam bath and allowing it to come to ambient temperature. Evaporation was complete when the mass of the oil remaining in the stainless beaker stabilized (≤0.03 g change in mass over a 30-min span on the steam bath). We recorded the final weight of the beaker and oil, and calculated the percent oil using the following formula:

% Oil = [(Weight of Beaker with Oil − Empty Beaker Weight)/Sample Weight] × 100(1)

The protein analysis (reference methods AOAC 992.15; AACC 46-30) was conducted by Medallion Labs (Minneapolis, MN, USA) and the sugar analysis (sugar by HPLC) was conducted by North Carolina Extension/North Carolina State University (Raleigh, NC, USA). 

### 4.5. Preparation of Nonsaponifiable Fraction (NSF)

We prepared 10% KOH/methanolic solution by dissolving 50 g KOH in 50 mL deionized water and bringing to volume of 500 mL with methanol. We prepared internal standard by mixing the epicoprostanol 5β-cholestan-3α-ol with heptane to a final concentration of 1mg/mL. After adding 25 µL of internal standard solution and 1 mL 10% methanolic KOH to each tube, the samples were saponified at 80 °C for 2 h using a Thermomixer (Eppendorf, Hamburg, Germany) with constant shaking at 500 rpm. Once cooled to ambient temperature, the nonsaponifiable fraction (NSF) containing free sterols was extracted with 1 mL of *n*-hexane. The hexane was pooled in a 1.5 mL microcentrifuge tube and the hexane was removed by evaporation in a fume hood overnight. To the residual, 20 µL of acetone was added to dissolve the sterols. After solvation, the compounds were converted to their trimethylsilyl ester by adding 10 μL of BSTFA and 10 μL of pyridine as catalyst. The derivatization mixtures were kept at room temperature for 30 min before GC/MS analysis.

### 4.6. GC/MS Analysis

2 µL of derivatization mixture was injected into an Agilent GC/MS (Agilent 6890 BC coupled with 5973 mass-selective detector (MSD)) (Agilent, Santa Clara, CA, USA). The GC was equipped with an Agilent DB-5Ms+DG narrow-bore capillary column (30 m × 0.25 mm × 0.25 µm with 10 m Duraguard). The injection mode was splitless, with helium carrier gas at a constant flow of 1.2 mL/min. The GC oven was initialed at 170 °C, held for 1 min, the temperature was ramped to 280 °C at 40 °C/min and held at 280 °C for 25 min. The MSD was in electron ionization (EI) mode, scan range was from 50–550 amu, temperature of the ion source was 230 °C, the quadrupole temperature was 150 °C, and the interface was 280 °C.

The GC/MS data was processed with ChemStation software (Version E.02.02.1431, Agilent, Santa Clara, CA, USA) and Automated Mass Spectral Deconvolution & Identification System (AMDIS) (National Institute of Standards and Technology, United States Department of Commerce, Washington, DC, USA). The sterol peaks were deconvoluted using AMDIS after baseline correction and identified by their relative retention time to cholesterol and comparison to the mass spectra from commercial mass database (NIST08 mass spectral library, http://nistmassspeclibrary.com/). The GC peak representing the sterol amount generated from total ion current (TIC) was integrated using the software default parameters [26,27].

## 5. Conclusions

Physiological maturity of *A. hypogaea* seeds is impactful to germination efficiency, and consequently, is of economic importance to the peanut industry. Immature seeds have reduced germination frequency, are more susceptible to disease, and require more financial inputs during manufacturing processes which are dependent on fat content or roasting performance. This study seeks to characterize phytosterol and isoprenoid content in seed components as a function of physiological maturation indicated by mesocarp color class.

Peanut hearts contained the highest percentage of phytosterols and isoprenoids by mass as compared to the other seed components. The stability of the peanut heart, or embryo, is critically important to the development and vigor of the seedling during germination and plant development. Tocopherols provide plants with antioxidant capacity and provide fluidity and flexibility to cell membranes. α-tocopherol concentration in the hearts changed dramatically across maturation classes, providing a possible causal agent for poor stress adaptability in immature seeds. Given the economic importance of seed efficiency and performance in all sectors of the peanut industry, α-tocopherol content in the seed embryo could be utilized as a biomarker to optimize harvest timing and profitability. In the subsequent study, we will investigate more details to evaluate using α-tocopherol as a biomarker for peanut harvesting, and will develop a sensitive and portable α-tocopherol detection method for peanut farmers.

Stigmasterol, one of the three end products in the phytosterol pathway in *A. hypogaea*, is a significant component by percentage of seeds. The seed coat, or skin, is known to be a major contributor of antioxidant capacity and other health benefits. The relatively high concentration of stigmasterol may contribute to the health benefits reported by the addition of peanut skins to various manufactured goods. Also, stigmasterol biosynthesis is important in plant physiology to deal with low and high temperature stresses, and plays a key role in innate immunity to combat biotic stresses. Since the main function of the seed coat is to protect the embryo, high stigmasterol content may be important for seed storage and germination.

## Figures and Tables

**Table 1:** Macronutrient Composition of *Arachis hypogaea* Seeds of Different Maturation Classes.

Pod Color	Fat (%)	Protein (%)	Sugar (%)
Orange	50.95	20.90	3.43
Brown	51.49	19.90	3.63
Black	51.96	18.00	4.42

Method error ± 0.33% fat, 0.648–0.798% protein, and −0.33–0.52% sugar.

**Table 2:** Phytosterol and Isoprenoid Composition of *Arachis hypogaea* Seed Hearts of Different Classes.

Sterols and Isoprenoids	Heart		
	Black	Brown ^1^	Orange
α-Tocopherol	0.24 ± 0.05	0.19 ± 0.02	0.09 ± 0.08
Cholesterol	0.19 ± 0.01	0.17 ± 0.01	0.19 ± 0.03
24-Methylenecholesterol	0.85 ± 0.06	0.86 ± 0.03	0.92 ± 0.13
Campesterol	17.42 ± 0.35	16.65 ± 0.53	16.78 ± 0.61
Stigmasterol	2.91 ± 0.25	2.87 ± 0.14	2.63 ± 0.1
β-Sitosterol	61.99 ± 0.48	62.03 ± 0.62	62.33 ± 2.15
Isofucosterol	9.75 ± 0.25	10.2 ± 0.15	11.01 ± 1.03
alpha-Amyrin	0.38 ± 0.05	0.38 ± 0.06	0.36 ± 0.23
Cycloartenol	2.35 ± 0.13	2.45 ± 0.14	1.95 ± 0.79
24-Methylenecycloartanol	1.95 ± 0.14	2.1 ± 0.27	1.65 ± 0.68
Citrostadienol	1.97 ± 0.11	2.12 ± 0.03	2.1 ± 0.19
Total (μg/mg)	8.68 ± 0.82	8.47 ± 0.61	10.25 ± 1.7

Sterol and isoprenoid content is AVE %; STD was calculated on each sterol for the sample; *n* = 5, ^1^ *n* = 4. Units are μg (sterol and isoprenoid)/mg (dry tissue).

**Table 3:** Phytosterol and Isoprenoid Composition of *Arachis hypogaea* Seed Kernels of Different Classes.

Sterols and Isoprenoids	Kernel		
	Black	Brown	Orange
Alpha-Tocopherol	0.63 ± 0.12	0.69 ± 0.38	0.24 ± 0.05
Cholesterol	0.21 ± 0.13	0.22 ± 0.11	0.19 ± 0.01
24-Methylenecholesterol	0.27 ± 0.2	0.86 ± 0.86	0.85 ± 0.06
Campesterol	12.14 ± 0.21	11.12 ± 1.42	17.42 ± 0.35
Stigmasterol	11.63 ± 0.49	11.61 ± 0.69	2.91 ± 0.25
β-Sitosterol	62.61 ± 1.9	58.7 ± 2.73	61.99 ± 0.48
Isofucosterol	10.7 ± 1.35	14.32 ± 1.42	9.75 ± 0.25
alpha-Amyrin	0.61 ± 0.43	0.65 ± 0.49	0.38 ± 0.05
Cycloartenol	0.7 ± 0.43	0.44 ± 0.29	2.35 ± 0.13
24-Methylenecycloartanol	0.33 ± 0.25	1.18 ± 0.84	1.95 ± 0.14
Citrostadienol	0.17 ± 0.12	0.21 ± 0.27	1.97 ± 0.11
Total (μg/mg)	1.04 ± 0.06	0.93 ± 0.08	8.68 ± 0.82

Sterol and isoprenoid content is AVE %; STD was calculated on each sterol for the sample; *n* = 5. Units are μg (sterol and isoprenoid)/mg (dry tissue).

**Table 4:** Phytosterol and Isoprenoid Composition of *Arachis hypogaea* Seed Coats of Different Classes.

Sterols and Isoprenoids	Seed Coat		
	Black	Brown	Orange
Alpha-Tocopherol	0.64 ± 0.12	0.71 ± 0.14	0.66 ± 0.14
Cholesterol	0.54 ± 0.07	0.57 ± 0.18	0.46 ± 0.25
24-Methylenecholesterol	0.13 ± 0.12	0.12 ± 0.04	0.12 ± 0.09
Campesterol	14.71 ± 0.91	14.71 ± 0.56	15.11 ± 0.63
Stigmasterol	3.76 ± 0.55	3.95 ± 1.04	4.57 ± 0.2
β-Sitosterol	75.78 ± 1.04	76.45 ± 1.19	75.19 ± 0.96
Isofucosterol	2.67 ± 1.73	2.09 ± 0.23	2.18 ± 0.29
alpha-Amyrin	0.99 ± 0.18	1.05 ± 0.14	0.89 ± 0.11
Cycloartenol	0.73 ± 0.61	0.24 ± 0.16	0.74 ± 0.57
24-Methylenecycloartanol	0.01 ± 0.01	0.04 ± 0.03	0 ± 0.01
Citrostadienol	0.06 ± 0.03	0.08 ± 0.04	0.06 ± 0.03
Total (μg/mg)	5.4 ± 0.62	5.51 ± 0.89	5.71 ± 0.86

Sterol and isoprenoid content is AVE %; STD was calculated on each sterol for the sample; *n* = 5. Units are μg (sterol and isoprenoids)/mg (dry tissue).

**Table 5:** The *p*-values of the pair-wise Student’s *t*-test among three developmental stages.

Sterols and Isoprenoids	Heart			Kernel			Seed Coat		
	BL-BR ^1^	BR-OR	BL-OR	BL-BR	BR-OR	BL-OR	BL-BR	BR-OR	BL-OR
α-Tocopherol	**0.041 ^2^**	**0.019**	**0.003**	0.367	0.285	0.327	0.206	0.300	0.395
Cholesterol	0.079	0.160	0.433	0.471	0.281	0.325	0.383	0.231	0.255
24-Methylenecholesterol	0.445	0.186	0.175	0.088	0.069	0.294	0.446	0.444	0.489
Campesterol	**0.028**	0.368	**0.039**	0.076	0.437	0.062	0.499	0.156	0.219
Stigmasterol	0.380	**0.017**	**0.025**	0.480	**0.027**	**0.013**	0.363	0.117	**0.008**
β-Sitosterol	0.460	0.389	0.369	**0.015**	**0.004**	0.148	0.183	0.051	0.191
Isofucosterol	**0.006**	0.078	**0.015**	**0.002**	**0.030**	0.127	0.241	0.301	0.276
alpha-Amyrin	0.409	0.442	0.408	0.442	0.153	0.174	0.282	**0.040**	0.161
Cycloartenol	0.169	0.118	0.148	0.154	0.338	0.090	0.061	**0.047**	0.484
24-Methylenecycloartanol	0.184	0.115	0.181	**0.032**	**0.018**	0.179	**0.047**	**0.032**	0.382
Citrostadienol	**0.023**	0.416	0.123	0.391	0.497	0.356	0.183	0.269	0.368
Total (μg/mg)	0.338	**0.040**	0.050	**0.021**	0.414	**0.030**	0.409	0.362	0.260

^1^ BL: black; BR: brown; and OR: orange. ^2^ The *p*-values less than 0.05 are in bold. Units are μg (sterol and isoprenoid)/mg (dry tissue).
